# Behavioural and structural interventions in cancer prevention: towards the 2030 SDG horizon

**DOI:** 10.1002/1878-0261.12805

**Published:** 2020-10-15

**Authors:** Jose M. Martin‐Moreno, Natalia Ruiz‐Segovia, Eduardo Diaz‐Rubio

**Affiliations:** ^1^ Department of Preventive Medicine & INCLIVA University of Valencia Spain; ^2^ Department of Prevention and Health Promotion Asociacion Española Contra el Cancer Madrid Spain; ^3^ Servicio Oncologia Medica Hospital Clinico San Carlos Madrid Spain

**Keywords:** Agenda 2030, behavioural interventions, cancer prevention, health in all policies, health promotion, noncommunicable disease prevention, structural interventions, sustainable development goals

## Abstract

Traditionally, the prevention of cancer (and other chronic diseases) has been considered primarily linked to personal responsibility, for which interventions must be based on health education information enabling individuals to make knowledge‐based decisions to improve their lifestyle. However, lifestyle is conditioned by environmental factors (including dimensions such as the context of economics, transport, urbanism, agriculture or education) that may render healthy behavioural choices either easier or, alternatively, impossible. This article reviews the conceptual underpinnings of the behavioural‐structural dichotomy. We believe that it is advisable to opt for multilevel strategies that take into account all the determinants of health, using structural and behavioural approaches, rather than only the latter, as has been done until now.

## Graphical abstract

Interventions in individual lifestyles have often been viewed as the main component of cancer prevention strategies. However, environmental factors may facilitate or impede healthy behaviours. The behavioural‐structural dichotomy of cancer prevention can only be resolved by incorporating the ‘Health in All Policies’ perspective at multiple levels (legislation, promotion of healthy choices, health support groups, health education).

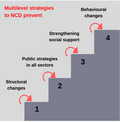

**Abbreviations**
EPHO: essential public health operations
NCD: noncommunicable diseases;
NGO: non‐governmental organisations
NICE: National Institute for Health and Care Excellence
SDG: sustainable development goals
UN: United Nations

## Introduction

This paper aims to contribute to the debate regarding the most effective approaches to cancer prevention are.

We will not go into a one‐by‐one systematic analysis of the specific interventions that have shown to be effective when implemented correctly, as these are available in other studies [1]. Instead, we intend to address a conceptual question: whether, in order to accomplish behaviour change, an individual approach based on persuasion is better than, worse than or complementary to addressing the social, economic and ultimately structural issues that can influence the success of cancer prevention.

In this sense, political debates in which political parties criticise each other by alluding to the level of interventionism (either too much or too little) are too common. Several regulations are occasionally put into practice (such as limiting sugar or energy drinks; monitoring and reducing saturated fat or salt in commercial menus; limiting the possible ways items such as tobacco or alcohol can be bought). This category of action is referred to as ‘structural interventions’. In this context, it is common to hear voices from conservative positions that express dissatisfaction with living in a state that ‘reduces individual freedom and responsibility’. The term ‘nanny state’ is also frequently used to criticise this exaggerated invasion of individual freedom, advocating that it would always be better to inform and from there, leave each person the choice of what or what not to do. At the other extreme, the absence of intervention and action to regulate products that involve potentially dangerous exposures or risk factors is criticised from the left wing as a lack of commitment to defend the interests of citizens, calling for detailed regulations to limit excesses by commercial interests. All of this must be put into the context of the 2030 Agenda for Sustainable Development adopted at the United Nations (UN), which has set specific, ambitious targets for reducing the disease burden associated with cancer and other chronic diseases, using all available tools at our disposal.

In this review article, we first address precisely ‘The 2030 horizon agenda for health and well‐being within the Sustainable Development Goals’ to contextualise the need to tackle prevention of cancer and other chronic diseases with the utmost determination. We then briefly review how understanding the determinants of health is essential to provide a basis for prevention and outline the lines of action effectively to prevent noncommunicable diseases (NCDs) in general, and cancer in particular. We then go on to describe the preventive strategies based on behavioural interventions, followed by the approaches based on structural actions. Finally, we propose the idea of combining strategies to maximise the impact of cancer prevention, ending with some conclusions and perspectives that we try to address in a spirit of hope.

## 1. The 2030 Agenda Horizon for Health and Well‐being within the Sustainable Development Goals

The UN promoted the Agenda 2030 for Sustainable Development as a blueprint and driver for the progress and sustainability of people and our planet. The agreement, signed in 2015, brings together the most critical global challenges of our socio‐political times and translates them into concrete objectives and goals intended to serve as a compass for the political actions of world governments.

Within the Agenda 2030 objectives, health and well‐being rank third on the list, reflecting the fact that this dimension is understood as a real priority for the UN. To ensure a sustainable planet, it is vital to guarantee the health of all the people who inhabit it. However, the world faces many diseases, risks and hazards that threaten universal health. Although in the unprecedented period of the COVID‐19 pandemic we are living through, communicable infectious threats are undoubtedly critical and a serious concern, we cannot forget that chronic NCDs lead to a higher rate of morbidity and mortality. To illustrate the strength of this statement, we can simply point out that NCDs kill some 41 million people worldwide every year. Deaths due to heart disease, chronic respiratory diseases, diabetes or cancer account for 71% of global deaths [2]. If nothing is done to prevent it, cancer will become the largest cause of death worldwide in the coming years.

To try to avert this, the UN proposes an ambitious but necessary target: to reduce premature mortality from NCDs by one‐third through effective preventive and treatment measures (target 3.4). Even though more than half of the global population dies from NCDs, most of these diseases are potentially preventable if we act on their risk factors in time [3]. Unhealthy diets, lack of physical activity and consumption of alcohol and especially tobacco are responsible for a substantial number of deaths from NCDs [2]. Unfortunately, failure to implement preventive interventions that we know are effective, is jeopardising the achievement of this target [4].

Motivated by all of the above, the UN Secretary‐General warned of the need to take immediate and urgent action involving a profound change in the strategies used if the objectives of Agenda 2030 are to become a reality for all by the agreed date [5]. Consequently, the World Health Organization’s Independent High‐Level Commission established practical recommendations to accelerate countries' progress towards achieving target 3.4 [6].

## 2. Understanding the implications of health determinants: a critical point to address the 2030 Agenda for SDGs

Since McKeown *et al*. [7] pioneered the term ‘health determinants’, Laframboise has developed a holistic model [8] that Minister Marc Lalonde later implemented through the document ‘New Perspectives on Canadian Health’ [9], identifying four categories that grouped the main factors of human health: biology (genetic inheritance, internal systems, ageing and development), environment (pollution, environment and social conditions), lifestyle (personal health decisions and choices) and organisation of healthcare (characteristics of the population's health system).

This categorisation allowed the concept of health to be segmented and organised, making it more manageable and easier to analyse. Additionally, it represented a crucial change in the conceptualisation of the term, as it raised biological, behavioural and environmental factors to the same level as the healthcare system, which had been the undisputed protagonist of health policies until then [9].

Later, other authors endorsed this scheme by providing updates that generated broader conceptual frameworks distinguishing different levels of intervention according to the health determinant (Table 1) [10, 11].

**Table 1:** Health determinants and interventions.

	Health determinants	Interventions	Actions
Level 1	Socio‐economic and environmental factors	Structural changes	Legislation, taxes, trade and environmental treaties/agreements
Level 2	Living and employment condition factors	Public strategies in all sectors	Changes in the environment that encourage healthy choices
Level 3	Social networks and community	Strengthening social support	Active health groups supporting each other
Level 4	Individual lifestyles	Behavioural changes	Health education and mentoring/counselling

## 3. Articulating lines of work for the prevention of NCDs in general, and cancer in particular

While the progress in the diagnosis and treatment of diseases has certainly enabled great achievements, prevention and public health actions have been shown to deserve top priority, as they are the most effective and efficient interventions to attain a healthy and dignified life [12, 13].

International institutions have provided quite a few resources as well as policy guidelines to steer our actions. The framework offered by the 10 ‘Essential Public Health Operations’ (EPHOs) of the World Health Organization has proven to be vital in addressing this challenge [14, 15].

Thus, the importance of population health surveillance (EPHO 1) is evident not only for infectious diseases and outbreaks (such as the COVID‐19 pandemic) but also for tracking the evolution of NCDs; the response to health hazards and emergencies is also a crucial function (EPHO 2). Moreover, we cannot forget the relevance of all other essential operations, such as communication and social mobilisation for health (EPHO 9); actions aimed at identifying and leading the implementation of immunisation and other preventive interventions (EPHO 10 and 5); health protection interventions that include environmental, occupational and food safety (EPHO 3); promoting the health and well‐being of the population with a practical approach tackling inequalities and broader social and environmental determinants (EPHO 4); appropriate health governance together with reliable infrastructure and financing to ensure resources and sustainability of public health interventions (EPHO 6 and 8); and responsibility to provide a competent workforce (EPHO 7).

## 4. Preventive strategies based on behavioural interventions

Traditionally, these types of interventions, aimed at promoting changes in individual lifestyles, have been at the forefront of strategic prevention priorities. However, in recent years, efforts to generate changes in health behaviour using these strategies have had limited success [3].

Behavioural interventions seek to improve the health of individuals through educational actions that provide them with the necessary information to make decisions that are beneficial to their health. These strategies appeal to individual responsibility and are based on the belief that people act in an eminently rational manner and consider the consequences of their behaviour before acting [16]. Behavioural interventions require limited political involvement and commitment. They tend to be well received by society and therefore seem to be less likely to alienate politicians and decision‐makers. In reality, the programmes that integrate these types of measures require a great deal of individual effort that is not accompanied by a meaningful public health impact [11, 17, 18].

The fact that behavioural interventions do not have all the success expected may be surprising. However, although they are based on sound behavioural change theories such as those of Bandura [19] or Becker’s health belief model [20], they generally ignore fundamental social factors, even those associated with the conceptual schemes themselves. Thus, Bandura [19] concludes that health is a social issue and not only an individual one and warns of the need to change social systems if we want to achieve significant outcomes on human health. There is ample evidence showing that social issues such as the economic crisis may have a definite impact on cancer prevention [21].

The misconceptions or myths on which policies founded exclusively on behavioural interventions are based, and the counter‐arguments explaining why these beliefs are not entirely correct, are set out in Table 2.

**Table 2:** False beliefs about the effectiveness of behavioural interventions and their counter‐arguments (modified from Marteau *et al*. 2015).

Beliefs	Counter‐arguments
Behaviour modification is common sense	The simplistic idea of ‘common sense’ and ‘intuition’ has created ineffective interventions that have cost resources and lost opportunities. In this type of scheme, it is considered that human behaviour is obvious and that introducing measures to modify it is simple, ignoring the scientific evidence of the disciplines that have studied this problem in depth. Change is complex and requires motivation and sustained support. In addition, it often happens that the health behaviours that need to be changed are shielded or supported by large industries that want to prevent those changes from happening.
Successful prevention is based on getting the right message across	Prevention strategies are not advertising campaigns, at least not exclusively. Preventive campaigns that have worked with successful messages and slogans have been multilevel strategies, in which advertising was only one part of a broader policy, not the only component. It is important that people understand the message and identify with it, but this is not enough to trigger a change in their behaviour.
Information and knowledge are sufficient to generate a change in behaviour	This model assumes that people smoke, drink alcohol, eat inappropriately or are not physically active because they lack information about the harmful effects of these behaviours. Therefore, if we tell them the negative consequences of their unhealthy habits, they should then change their behaviour to a healthier one. But it does not really work like that. Marteau *et al*. [25] worked with focus groups of young women who were asked about this premise, and the participants conveyed that they knew the benefits of eating healthier but that there were contextual factors that made it difficult for them to follow the advice. Information is not enough to produce changes in behaviour.
People always act rationally in their decisions	If people were always acting rationally, when they receive information about what is good for their health, they should change their behaviour,.. but clearly, they do not. Sometimes health behaviour is on a less conscious level and is driven by automatic processes influenced by the environment. However, it is also not true that people always act irrationally; they have their own reasons for behaving in a certain way. We must evaluate and take into account the functionality of their behaviours, their reasons and motives to act within the context in which they live [36, 37].
The individual approach is sufficient and adapted to the person	In reality, pure behavioural interventions do not take into account the life context of the people where the health behaviour takes place [18]. Human behaviour is influenced by environmental stimuli and the architecture of the environment, and if we increase the availability of a healthy option within an environment, we increase the chances that people will choose that option [25].

## 5. The option of strategies based on structural interventions

As discussed above, an individual's behaviour and ability to make healthy decisions depends mainly on the factors that characterise their environment and life, including economic power and social status [22, 23]. The circumstances in which individuals grow up, live and age have an enormous influence on their health and are a result of the social, political and economic contexts in which society is embedded. This explains most of the inequities in access to universal healthcare that the SDG 2030 Agenda aims to mitigate. Developing countries have a higher probability of exposure to risk factors and a lower capacity for disease prevention mechanisms. These countries account for more than 85% of the world's NCD cases [2].

Structural approaches aim to change the architecture of the process by which our choice is made [18]. This is achieved by encouraging healthy options through changes in the context in which they take place, thus making the healthy choice the easiest to make, regardless of the person's education and socio‐economic level [11]. Behaviour is not reduced exclusively according to what individuals think or do in isolation. It is well known that relationships between people and their environment conceptualise behaviour [18].

Structural strategies focus on the environmental factors that influence risk behaviour, rather than on the individual features of the person who carries it out [24]. Some of these factors are listed here.

### Availability

Improving the readiness of the healthy option increases the ease of use and the likelihood of choice [25].

### Design

The design of a product influences our perception and how we relate to it. Generally, we approach products that we perceive as exciting and rewarding stimuli and move away from harmful and threatening ones. Altering the existing associations with risk factors and creating new ones can be a suitable way for people to change their behaviour in the context of different environmental signals [25].

### Price

Epstein and collaborators [26] conclude in their review that changing the price of the product has a more significant effect than informational and educational interventions in getting most people to make the healthy choice. However, even accepting that, there are undoubtedly superior benefits from combining both interventions.

## 5. Combining strategies to maximise the impact of cancer (and other NCDs) prevention

First, we must recognise that, despite the established limitations of exclusive behavioural strategies to prevent NCDs, these interventions have come to dominate almost entirely the health policies of most governments and organisations. Traditionally, people have been held responsible for their health, citing their choice of ‘lifestyle’ and their level of exposure to NCD risk factors. This has been quite common and, in particular, has been more so in governments of neo‐liberal ideology, which by nature are prone to a lower level of intervention and a higher transfer of responsibility to the individual. Furthermore, placing the responsibility for their actions on the individuals for changing their choice for a healthier one may be convenient for the decision‐makers. In fact, this exempts the decision‐makers from entering into conflict with powerful companies or corporations that potentially promote products or services posing a health risk, while also providing the comfort of avoiding implementing legislative changes that could generate social grievances.

All of the above do not imply that we should disregard the importance of informing, forming, educating and promoting healthy behaviours. Rather, this must be done with the understanding that people's health does not depend solely on their individual choices but that the options are also conditioned by positions that governments and public and private entities must assume to promote population health. For example, the argument that people choose their food can dilute the responsibility of instituting relevant measures in production, marketing and promotion of food that influence the choices of individuals. The state must create a context that controls the food environment, simplifying and facilitating healthy options [27].

Among the most relevant initiatives in this area of cancer prevention are the successive versions of the *European Code Against Cancer*. This code lists a set of recommendations for individuals on how to reduce cancer risk and focuses almost exclusively on the information dimension to improve healthy behaviour. Only in the 4th edition (the latest one) did the code go beyond individual action, clearly stating that it is essential to introduce ‘public health policies and actions by national governments (when exposure is eliminated or reduced by effective and equitably accessible preventive measures at the population level)’ [28].

Leadership in health can adopt multiple positions within a continuum that ranges from observation, monitoring and persuasion for the healthiest possible behaviour at one end, to regulation through restriction or elimination of products at the other [27]. Governance for health must come from governments, non‐governmental organisations (NGOs), public and private entities, and the entire community, including every individual. Governing bodies must act for the benefit of people's health, using strategies that are effective and discarding those that are not [27]. To this aim, it is recommended that governments allow themselves to be advised by entities with experience and knowledge to formulate health strategies. In this regard, some governments are beginning to consider the evidence of behavioural science to reformulate their strategies, thus adopting a more realistic view of human behaviour [16].

Finally, we believe it is vital that the initiatives mentioned above are structured and reflected through cancer control plans, promoting the complementary synergy of interventions to achieve more of an impact [29].

## 7. Conclusions and perspectives, in a spirit of hope

To improve cancer prevention and reduce morbidity and mortality from cancer (and other NCDs), and to address target 3.4 of the 2030 Agenda of the Sustainable Development Goals approved by the United Nations, it must be recognised that health behaviour takes place in a social context. Prevention policies must take this into account [18]. If we want to eradicate NCDs, it is essential to evaluate and change the environment, involving all sectors of society (e.g. finance, production, transport, consumption, urban planning, education, agriculture). This can be achieved with a cross‐cutting approach that brings everyone together in a joint policy. When the socio‐economic context, the environment and the health system work together, we will achieve primary prevention [30].

NICE guidelines recommend that individual interventions be complemented by community and organisational strategies, taking into account the social and cultural contexts of the population and ensuring equity of access to healthy living. Behaviour modification is more likely to be sustained over time when multilevel interventions [17] that integrate organisational, community and individual actions are used, allowing for structural and behavioural strategies [31].

Table 3 shows practical examples that have been effective in bringing about changes in the health of the population through the implementation of measures at all levels, in this case for tobacco control policy and healthy nutrition.

**Table 3:** Implementation of multilevel measures for tobacco control and nutrition policy.

	Goals	
	Tobacco control [10]	Healthy nutrition [38]
Levels	Price increases (Level 1)	Analysis of health and quality of life (Level 1)
	Bans and smoke‐free spaces (Level 2)	Analysis of behaviour and environmental risk factors (Level 2)
	Support for communities to ensure that tobacco is not sold to minors (Level 3)	Analysis of determinants of risk behaviours (Level 3)
	Education for citizens about the harmful effects of tobacco (Level 4)	Intervention mapping and implementation (Level 4)

Another example is a multilevel strategy that, seeking to adopt healthy behaviours through healthy nutrition, proposes specific measures such as adjustment of healthy food prices, especially for the most vulnerable groups (Level 1); incentives for the agricultural sector to produce more nutritious food at a lower cost to the consumer (Level 2); neighbourhood food cooperatives (Level 3); and improved labelling and nutrition education to encourage individual behaviour change [10].

Although assessment of the results achieved with multilevel strategies is crucial to evaluate the effectiveness and efficiency of programmes, the multivariate nature of this type of intervention makes it difficult analytically to control the specific influence of exposure variables and confounding factors. In other words, we face a methodological challenge for evaluations, as it is challenging to identify the impact of particular measures (behavioural and structural) within the framework of the multilevel strategy. To assess the effects of the actions on the population more accurately, we will have to make precise definitions of the predictor variables, the intermediaries and the results of interest, operationalising the behavioural changes to be measured [32].

As an alternative to the quantitative assessments that are particularly difficult to disentangle here, qualitative assessment techniques can be better adapted and more appropriate for this type of complex evaluations [33]

In the end, the essential point is understanding the importance of incorporating the ‘Health in All Policies’ perspective [34]. This entails involving the whole of society (public authorities and the private sector, scientific entities, and the community as a whole). Everybody should commit to good corporate governance, placing health above economic benefits, and highlighting the importance of incorporating all sectors of the population to achieve the objectives, understanding the underlying causes to obtain fair outcomes [35]. These points, together with information, education from schools and empowerment of people, constitute the way forward for effective primary prevention of cancer and other chronic diseases.

## Conflict of interest

The authors declare no conflict of interest.
